# Copolymers and Hybrids Based on Carbazole Derivatives and Their Nanomorphology Investigation

**DOI:** 10.3390/nano9020133

**Published:** 2019-01-22

**Authors:** Stefania Aivali, Sofia Kakogianni, Charalampos Anastasopoulos, Aikaterini K. Andreopoulou, Joannis K. Kallitsis

**Affiliations:** 1 Department of Chemistry, University of Patras, University Campus, Rio-Patras GR26504, Greece; aivali.s@upnet.gr (S.A.); skakogianni@upatras.gr (S.K.); xanastasop@upatras.gr (C.A.); andreopo@upatras.gr (A.K.A.); 2 Foundation for Research and Technology Hellas/Institute of Chemical Engineering Sciences (FORTH/ICE-HT), Platani Str., Patras GR26504, Greece

**Keywords:** vinyl functionalized PCDTBT, PCDTBT: PC_71_BM blends, fullerenes, hybrid semiconducting polymers, nanomorphology

## Abstract

Oligomers of the low-band-gap PCDTBT polymer, based on either 3,6 or 2,7 carbazole units, were modified with vinyl ω-chain end functionalities. The vinyl-functionalized oligomers were used as comonomers in free radical polymerizations with quinoline-based monomers such as 6-vinylphenyl-(2-pyridinyl)-4-phenyl-quinoline (vinyl-QPy), and 6-vinylphenyl-(2-perfluorophenyl)-4-phenyl quinoline (vinyl-5FQ). The co-polymeric materials bearing the vinyl-QPy moiety were developed as potential compatibilizers in polymer electron donor–fullerene acceptor blends for non-covalent interactions with the fullerene part. The co-polymeric materials bearing the vinyl-5FQ moiety were developed for the covalent attachment of carbon nanostructures; specifically, PC_61_BM. Both copolymers and hybrids, after thorough purification, were characterized in terms of their spectroscopic and optical properties as well as their ability to form nanophased separated films as such, or as additives at various percentages into PCDTBT: PC_71_BM blends.

## 1. Introduction

In recent years, organic photovoltaics (OPVs) based on conjugated polymers have come to be considered a promising route for renewable energy production devices due to their advantages, such as their low cost, flexibility, light weight and their offering facile coverage of large-scale areas of various shapes, compared to silicon-based solar cells [1,2,3]. So far, organic solar cells composed of a binary mixture of a polymeric electron donor and a fullerene derivative as the electron acceptor (Bulk Heterojunction OPVs) have achieved power conversion efficiencies (PCEs) of over 10% [4,5,6,7]. Some of the most promising systems for these devices are the P3HT:IC_60_BA [8,9,10] and the PCDTBT:PC_71_BM [11,12], from both efficiency and stability points of view. To further improve the efficiencies of OPVs, there are many factors that need to be considered, such as the development of efficient electron donors, combined with the proper electron acceptors [13,14,15] and the control and stabilization of the morphology of the active layer [16,17,18,19,20].

The functionalization of the semiconducting polymers used as electron donors has opened the route to the formation of more effective nanophase-separated active layers, which, moreover, could be used to create alternative copolymers [21,22,23,24] for the purpose of affording not only efficient, but also stable, OPVs under a variety of time and environmental conditions. The introduction of various functional units onto semiconducting polymers, either as side chains or at the α- and/or ω- end positions of the polymeric chains, allows a variety of approaches for the formation of complex macromolecular architectures or even hybrid structures. Such modifications on the semiconducting polymeric chains enable the covalent attachment of a semiconducting polymer onto carbon nanostructures, creating hybrid materials that are expected, and in many cases have indeed revealed, combined properties, altering and fine-tuning the morphology and nanophase separation of the pure component blends [25,26,27,28,29,30]. Another approach for the formation of hybrid structures is the development of non-covalent interactions between a molecular dispersant (in most cases a pyridine unit) and fullerene species. Such interacting species can be incorporated either into copolymers, or onto the molecular additives of the active layer binary blend. Ikkala et. al. reported the synthesis of a rod-coil copolymer bearing PS/P4VP units, which interacts with the fullerenes towards the formation of self-assembling domains [31]. In that study, the electron-donating pyridine moiety forms charge-transfer complexes with the electron-accepting C_60_ molecules in both bulk and spin-casted films, leading to improved nanophase separation. The research group of Hadziioannou [32] introduced a P3HT-b-P4VP block copolymer as a nanostructuring agent in the polymer/fullerene blend, and a significant enhancement of the efficiency was observed. The addition of pyridine units directly at the end or side positions of polymeric electron donors most probably leads to their incorporation at the donor/acceptor interface, thus influencing the crystallinity of the fullerene species and hence increasing the power conversion efficiency of the device [33,34].

Herein, copolymers and hybrids based on low-band-gap oligomers of the poly (N-9′-heptadecanyl-2,7-carbazole-alt-5, 5-(4′,7′-di-2-thienyl-2′,1′,3′-benzothiadiazole)), PCDTBT family are reported. In particular, vinyl end functionalized low-band-gap PCDTBT oligomers are used as macro-co-monomers in free radical polymerizations with two different electron-accepting quinoline-bearing vinyl monomers. In the first case, copolymers bearing the phenyl- (2-pyridinyl) -4-phenyl-quinoline (QPy) moiety were developed as potential stabilizers/compatibilizers of PCDTBT:PC_71_BM binary blends in order to employ the non-covalent interactions of the fullerene part with the quinoline-pyridine units. In the second case, copolymers bearing the phenyl- (2-perfluorophenyl) -4-phenyl- quinoline (5FQ) moiety were developed, since the perfluorophenyl groups can covalently attach onto the sp^2^ hybridized surface of carbon nanostructures. This latter approach was recently demonstrated by our group, and allows the direct connection of the semiconducting species onto the carbon nanostructures, creating hybrid materials in which the properties of the constituent organic and carbon nanostructure entities are influenced and/or combined [35].

## 2. Materials and Methods 

### 2.1. Materials

PCDTBT was purchased from Solaris Chem. Inc. (QC, Canada) PC[60]BM 99% and PC[70]BM 99% were purchased from Solenne b.v. Groningen, The Netherlands. Tetrahydrofuran was purchased from Aldrich (Sigma-Aldrich Chemie GmbH, Taufkirchen, Germany) and was distilled with benzophenone and metallic sodium (THF (dry)). All other solvents and reagents were purchased from Aldrich (Sigma-Aldrich Chemie GmbH, Taufkirchen, Germany ) or Alfa Aesar (Thermo Fisher (Kandel) GmbH Postfach, Karlsruhe, Germany) and were used without further purification unless otherwise stated. 6-Vinylphenyl-(2-pyridinyl)-4-phenyl-quinoline (vinyl-QPy) [36], 6-vinylphenyl-(2-perfluorophenyl)-4-phenyl-quinoline (vinyl-5FQ) [37], 4,7-di-2-thienyl-2,1,3-benzothiadiazole (di-thien-BTZ) [38,39], 4,7-di-(5-bromothiophene-2-yl-2,1,3-benzothiadiazole (di-Br-di-thien-BTZ) [40], 2,7-dibromo-*N*-heptadecan-9-yl-carbazole (di-Br-HD-carbazole) [41], 2,7-di(4,4,5,5-tetramethyl-1,3,2-dioxaborolan)-N-heptadecan-9-yl-carbazole (di-boronic ester-HD-carbazole) [41], 3,6-dibromo-*N*-2-ethylhexyl-carbazole (di-Br-EH-carbazole) [42], 3,6-bis(4′,4′,5′,5′-tetramethyl-1′,3′,2′-dioxaborolan-2′-yl)-N-9″-(ethylhexyl) carbazole [43] and the catalyst palladium (II) tetrakis triphenyl phosphine (Pd(PPh_3_)_4_) [44] were synthesized according to published procedures.

### 2.2. Characterization 

^1^H, ^19^F Nuclear Magnetic Resonance (NMR) spectra were recorded on a Bruker Advance (Bruker BioSpin GmbH, Magnet Division, Karlsruhe, Germany) DPX 400.13, 376.5 and 40.55 MHz spectrometer, respectively, with CDCl_3_ as solvent containing TMS as internal standard. 

Gel permeation chromatography (GPC) measurements were carried out using a Polymer Lab chromatographer (Agilent Technologies, Santa Clara, CA, USA) equipped with two PLgel 5 μm mixed columns and a UV detector, using CHCl_3_ as eluent with a flow rate of 1 mL/min at 25°C calibrated versus polystyrene standards.

Attenuated Total Reflectance (ATR) spectra were recorded on a “Bruker Optics’ Alpha-P Diamond ATR Spectrometer of Bruker Optics GmbH” (Ettlingen, Germany).

Thermogravimetric analysis (TGA) was carried out on ~8 mg samples contained in alumina crucibles in a Labsys TM TG apparatus of Setaram (Caluire, France) under nitrogen and at a heating rate of 10 °C/min

UV-Vis spectra were recorded using a Hitachi U-1800 spectrophotometer (Hitachi High-Technologies Europe GmbH, Mannheim, Germany). Continuous wave photoluminescence was measured on a Perkin Elmer LS50B spectrofluorometer (Waltham, MA, USA). All UV-Vis and PL measurements were performed in air using quartz cuvettes and flat quartz substrates for the examination of solutions and films, respectively.

Transmission electron microscopy (TEM) measurements were performed on a JEOL JEM2100 (Tokyo, Japan) operating at 200 kV. Sample preparation for TEM examination involved the drop-casting of diluted in *o*-DCB solutions and the removing the excess of the solvent with filter paper onto 3 mm carbon coated copper grids (Electron Microscopy Sciences, Hatfield, PA, USA) and were dried in air for 2 days.

The detailed experimental procedures for all the synthesized materials are given in the Supplementary Materials section.

## 3. Results

### 3.1. Synthesis of Functionalized Vinyl PCDTBT-Based Macromonomers

**Scheme 1:**
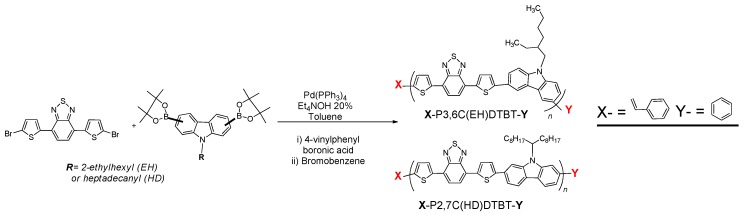
General synthetic procedure for ω-vinyl functionalized PCDTBTs.

Oligomers of the poly(carbazole-*alt*-benzothiadiazole)-PCDTBT family were synthesized via Suzuki cross-coupling polymerization reaction and were modified in situ at the *ω-*end positions of the polymer’s backbone with functional groups (see Scheme 1). To investigate the influence of the carbazole derivative onto the polymer’s properties, we used either the 3,6 or the 2,7-carbazole derivative. Firstly, in order to get PCDTBT oligomers of increased solubility, we employed the 3,6-carbazole monomer, which leads to more soluble and less rigid polymeric chains [45,46]. The desired monomer of di-Br-di-thien-BTZ was synthesized according to the procedures in the literature [39,40,41]. The final PCDTBT-type oligomers were synthesized via Suzuki cross-coupling using the catalyst:base system Pd(PPh_3_)_4_:Et_4_NOH 20% [47]. At the last step of the polymerization reaction, 4-vinylphenylboronic acid and bromobenzene were added successively to the mixture to introduce polymerizable double bonds at the end position of the polymer and a phenyl group at the other end, creating monofunctional, “active” polymers that could be used as macromonomers in free radical polymerizations (FRP) combined with other vinyl-monomers. Due to structural differences of the 3,6- carbazole to the 2,7- carbazole monomers, and in order to control the molecular weight leading to oligomers that are soluble enough and could be used afterward in free radical polymerizations, different polymerization periods were employed in these two cases, as shown in Table S2. In both cases, the crude vinyl functionalized PCDTBT-type oligomers were precipitated into methanol and further purified and fractionated via Soxhlet extraction with acetone and cyclohexane to remove salts, catalyst and any residual monomers [48] and finally tetrahydrofuran, so as to receive polymeric fractions, useful for the next FRP steps. To receive the higher molecular weights of both 3,6- and 2,7- vinyl functionalized polymers and at the same time to keep the double bonds “alive”, the insoluble fraction obtained after the THF extract, was heated at temperatures no higher than 80 °C with chlorobenzene, (CB), for 6 h and then filtrated at 60 °C.

**Figure 1:**
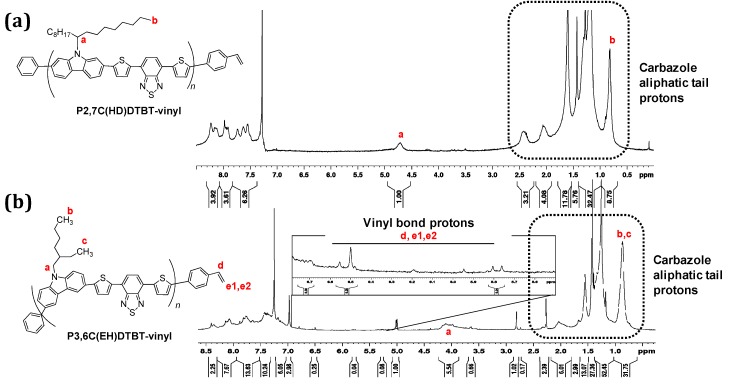
(**a**) ^1^H NMR spectrum of P2,7C(HD)DTBT-vinyl in CDCl_3_ and (**b**) ^1^H NMR spectrum of P3,6C(EH)DTBT-vinyl in CDCl_3._

**Figure 2:**
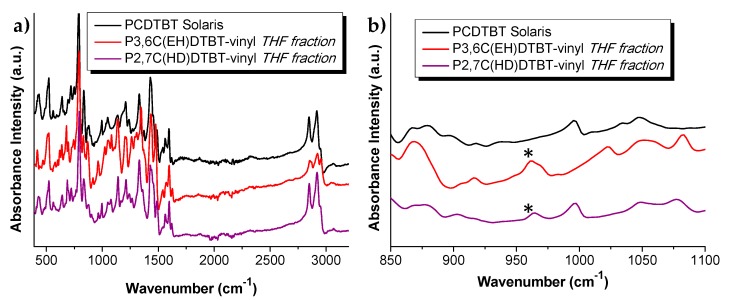
(**a**) ATR spectra of commercial PCDTBT, P3,6C(EH)DTBT-vinyl and P2,7C(HD)DTBT-vinyl THF fractions. (**b**) Enlarged region of the ATR spectrum from 850 to 1100 wavenumbers. The asterisks (*) in Figure 2b, indicate the peaks owing to the *ω*-end double bonds.

The successful introduction of the end double bonds was confirmed through ^1^H NMR (Figure 1 for the THF fraction of the oligomers) and ATR (Figure 2 and Figure S1 for the THF and the CB fractions of the oligomers). From the ^1^H NMR spectrum of P3,6C(EH)DTBT-vinyl, peaks at 5.26, 5.76 and 6.72 ppm are attributed to the double bonds at the end position of the polymeric chain. Unfortunately, in the ^1^H NMR spectrum of P2,7C(HD)TBT-vinyl the respective peaks were not detectable due to the higher molecular weight of the polymer and its lower solubility in CDCl_3_. Both P3,6C(EH)DTBT and P2,7C(HD)DTBT end-vinyl functionalized oligomers’ ATR spectra were compared to the commercial PCDTBT. Besides the characteristic bands of the PCDTBT backbone, a band at ~960cm^−1^ appears for vinyl-functionalized macromonomers, which is attributed to the end double bonds.

### 3.2. Synthesis of PCDTBT-Quinoline Copolymers 

**Scheme 2:**
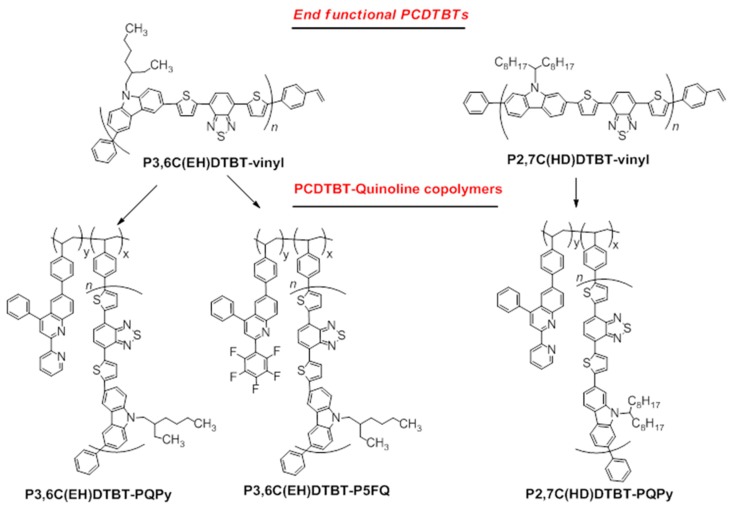
Synthesis of PCDTBT-quinoline-based copolymers.

The above-synthesized vinyl-functional oligomers of the poly(carbazole-*alt*-benzothiadiazole)-PCDTBT family were employed in free radical polymerizations (FRP) with vinyl-quinoline-based monomers, as shown in Scheme 2.

The monomer 6-vinylphenyl-(2–pyridinyl)-4-phenyl-quinoline (vinyl–QPy) was copolymerized via FRP with both the 3,6-carbazole- and the 2,7-carbazole-bearing PCDTBT macromonomers, affording the P3,6C(EH)DTB-PQPy and P2,7C(HD)DTBT-PQPy copolymers, respectively. The quinoline–pyridine unit was selected in order to create copolymers that could interact non-covalently with the fullerene electron acceptors of the active layer blends.

On the other hand, copolymers bearing PCDTBT and the perfluorophenyl quinoline unit were synthesized using the 3,6-carbazole-carrying PCDTBT macromonomer (P3,6C(EH)DTBT) and the 6-vinylphenyl-(2-perfluorophenyl)-4-phenyl quinoline monomer. The perfluorophenyl group was specifically selected in this case, since it can be transformed to an azide and covalently attach onto fullerene species, affording hybrid copolymeric materials [35]. Although the copolymer P3,6C(EH)DTBT-P5FQ was successfully synthesized, attempts to prepare the respective copolymer based on the 2,7 carbazole derivative P2,7C(HD)DTBT were unsuccessful, since the product could not be isolated in workable yield.

In all cases, the vinyl-functionalized PCDTBT macromonomers were copolymerized via FRP with the respective vinyl-quinoline derivatives at a 1:8 weight ratio (PCDTBT macromonomer:vinyl-quinoline derivative) using AIBN as the initiator in a solvent mixture of THF/DMF, forming the desired PCDTBT-quinoline copolymers. The crude products were extensively purified by washing with various solvents such as methanol, ethyl acetate and diethyl ether in order to ensure the successful removal οf any unreacted monomers. In Table 1, the theoretical and experimental ratio of PCDTBT:quinoline for the copolymers **M1**–**M5** are presented.

**Figure 3:**
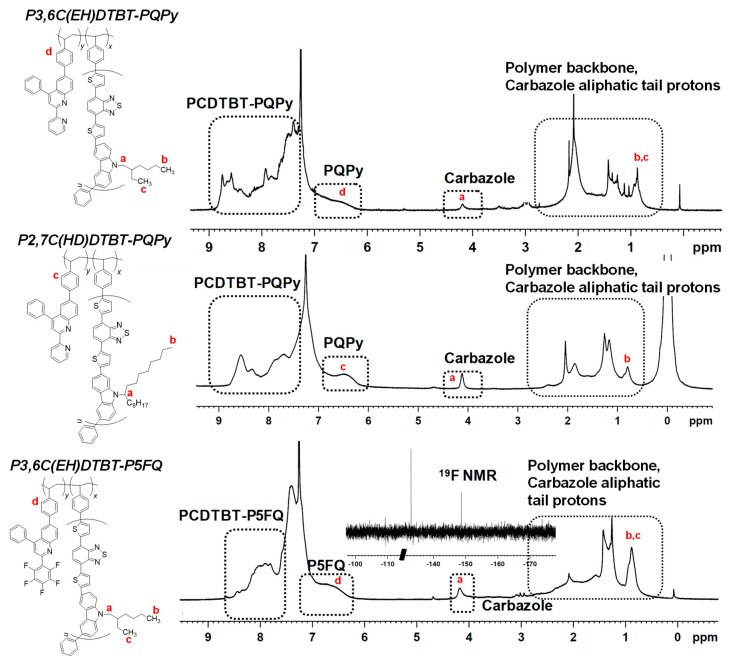
^1^H NMR spectra of the PCDTBT-Quinoline copolymers in CDCl_3._

From the ^1^H NMR spectra of the copolymers, (Figure 3), the absence of any peaks that could be attributed to double bonds in combination to the observation of well-resolved characteristic peaks of the quinoline groups at ~6.9–6.3 ppm and of the PCDTBT moiety at ~4.2 ppm, confirmed the successful copolymerization of the two moieties. Aside from these, broad peaks attributed to both the quinoline and PCDTBT blocks of the copolymers at ~7.3–8.8 ppm can be observed. From integration of the peak at 4.2 ppm, owing to the ethylhexyl methylene protons or the heptadecanyl methane protons of the carbazole units, respectively, and of the peaks at 6.3–6.9 ppm owing to aromatic protons of the quinoline monomers [35,37], the ratio of PCDTBT to the quinoline blocks was calculated. For the P3,6C(EH)DTBT-P5FQ copolymer, ^19^F NMR analysis was also performed, which is depicted in the insert of Figure 3, confirming the three different types of fluorine atoms present at the final copolymer due to the P5FQ block. The ATR spectra of the copolymer, together with the spectra of the initial monomers, (Figure S2), also confirm the absence of double bonds and the coexistence of peaks owing to both the copolymer blocks.

In Table 1, the PCDTBT-quinoline theoretical weight percent ratio together with the experimentally found percent ratio calculated from ^1^H NMR spectra are presented. It is obvious that in some cases (e.g., copolymers **M1, M2, M5**), a fairly good comparison between the theoretical and experimental weight percent ratios of PCDTBT to QPy units is observed. In other cases, (e.g., copolymers **M3, M4**), a significant discrepancy is observed, which can be attributed to the possible formation of vinyl-quinoline oligomers that are effectively removed during the purification process as supported by the GPC analysis using different UV detection wavelengths, namely, 254 and 540 nm, where the quinolines and the PCDTBT segments are absorbed, respectively. SEC characterization was performed for all synthesized copolymers with a UV detector both at 254nm and 540 nm wavelengths, at which the quinoline and the PCDTBT blocks, respectively, present their maximum absorbance. The results shown in Table 2 provide the starting molecular weights of the PCDTBT macromonomers and the molecular weights obtained for the corresponding copolymers. All copolymers presented similar Mn and Mw values, as detected at the two wavelengths of 254 and 540 nm, respectively, indicating successful copolymerization and excluding any scenario of homopolymer blends instead of copolymers. We should also point out that the molecular weight of the vinyl-PCDTBT derivative plays an important role on the molecular weight of the final PCDTBT-quinoline copolymer. More specifically, in the case of oligomers isolated from the THF- fractions, the final ratio of the respective block into the copolymer was found low. However, due to the complexity of the structure and specifically to the bearing quinoline pyridine units that probably interact with the columns’ packing material, the characterization through SEC could only provide indicative results. GPC characterization of PCDTBT-P5FQ copolymer and vinyl PCDTBT using the two detectors at 254 and 540 nm is presented in Figure S3.

The results included in Table 1 and Table 2 can be used for the estimation of the molecular weight of the copolymers. More specifically, from the proton integrals and the calculation of the copolymers’ MW from the ^1^H NMR spectra, it is obvious that in all the copolymers, at least one PCDTBT macromonomer is attached to each polymeric backbone, while in some cases, even two PCDTBT macromonomers are present. This is also supported by the fact that the GPC traces detected at 254 and 540 nm are similar, showing that at least one PCDTBT macromonomer is combined with the quinoline units in the copolymers. Based on that assumption, and taking into consideration the ratio of PCDTBT to the quinoline units calculated from the ^1^H NMR spectra, the real MWs of the copolymers can be estimated, and they are in the range of 38 to 49 kDa. These are an order of magnitude higher than the copolymers’ MWs found via GPC, which is attributed to the strong interactions of the quinoline moieties with the stationary phase of the GPC columns.

### 3.3. Optical Characterization of Copolymers

**Figure 4:**
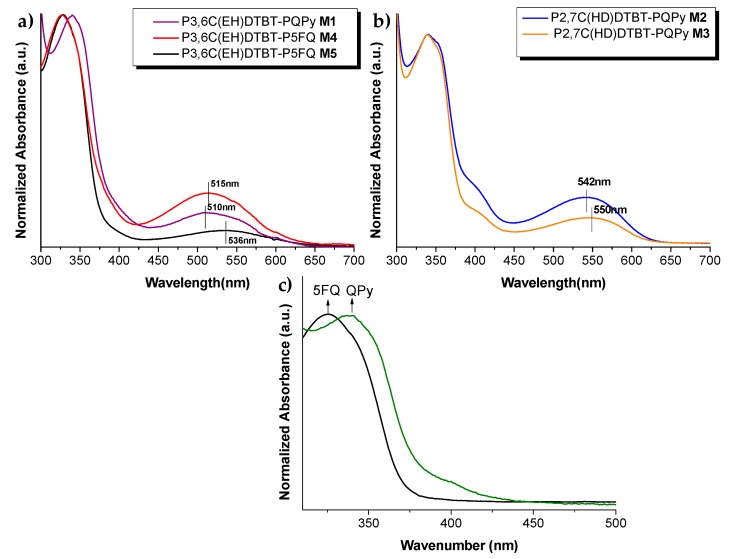
Normalized absorption spectra of (**a**) 3,6-carbazole-based copolymers **M1, M4, M5** in *o*-DCB solutions and their maximum absorbance; (**b**) 2,7-carbazole-based copolymers **M2, M3** in *o*-DCB solutions and their maximum absorbance; (**c**) absorption spectra of 6–vinylphenyl-(2- perfluorophenyl)–4–phenyl quinoline and 6-vinylphenyl-(2–pyridinyl)-4-phenyl-quionoline in *o*-DCB solutions.

The optical properties of the different PCDTBT copolymers synthesized (**M1–M5,** see Table 2) and of the starting vinyl-quinoline monomers were investigated in solutions, and the obtained results are given in Figure 4a–c, respectively. In Figure S4, the absorbance spectra of vinyl PCDTBT macromonomers in *o*-DCB solutions are also presented. The UV-Vis spectra of the copolymers in *o*-DCB solutions show the coexistence of the quinolines’ absorption bands and of the PCDTBT blocks. Copolymers bearing the perfluorophenyl-quinoline moiety present an absorption maximum at 330 nm, whereas copolymers bearing the pyridinylphenyl-quinoline show a red-shifted absorption band at 340 nm. Also, a second absorption maximum was observed in the copolymers’ case at 500–550nm, owing to the PCDTBT block. A difference in the absorption maximum was observed due to the different molecular weights of the PCDTBT macromonomer employed for the copolymers’ synthesis **M1–M5.** Particularly, for higher MW of the initial PCDTBT macromonomer, a more red-shifted absorption peak at ~550 nm is observed, as shown in Figure 4.

### 3.4. Blends of PCDTBT:PC_71_BM with PCDTBT-PQPy Copolymers 

The synthesized copolymers were studied as additives in mixtures of PCDTBT:PC_71_BM 1:1 wt% ratio. In Table 3, the composition of the blends obtained from o-DCB solutions are presented.

**Figure 5:**
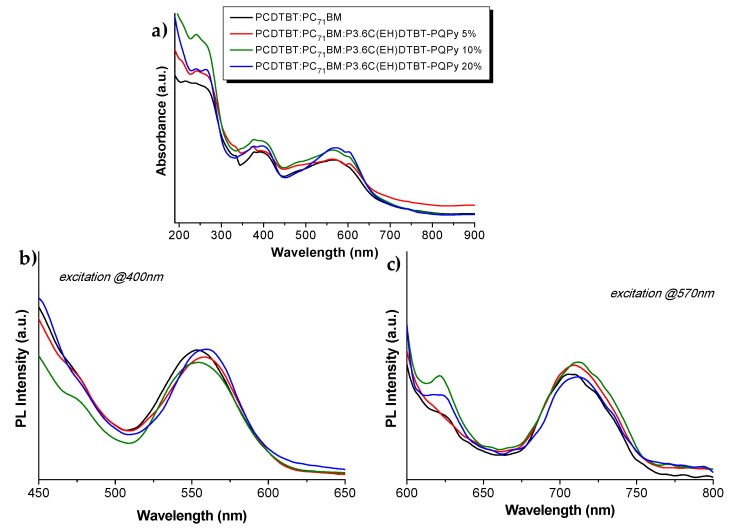
(**a**) UV-Vis specta; (**b**) PL spectra upon excitation at 400 nm; and (**c**) PL spectra upon excitation at 570nm of the PCDTBT:PC_71_BM:P3,6C(EH)DTBT-PQPy blends of spin-coated films.

The UV-Vis spectra in film form of the blends 2-4 (Figure 5) presented the same absorbance bands as the PCDTBT:PC_71_BM blend. Upon excitation at 400 nm, no remarkable difference could be observed. Upon excitation at 570 nm, a slightly increased photoluminescence peak was observed for blends bearing 5% and 10% of the copolymer, whereas a lower photoluminescence peak was observed for the blend bearing 20% of the copolymer.

**Figure 6:**
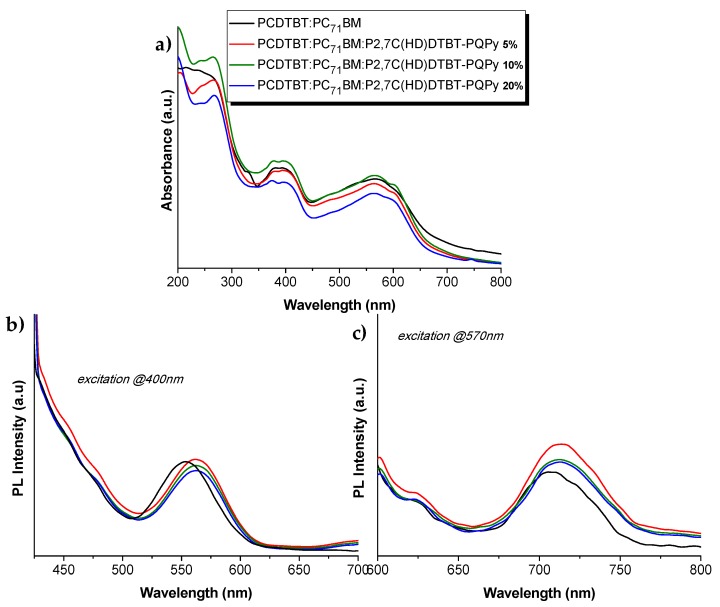
(**a**) UV-Vis specta; (**b**) PL spectra upon excitation at 400 nm; and (**c**) PL spectra upon excitation at 570nm of the PCDTBT:PC_71_BM:P2,7C(HD)DTBT-PQPy blends of spin-coated films.

The UV-Vis and PL spectra of blends 5–7 in spin coated films are shown in Figure 6, where no great differentiations are observed for the blends bearing the copolymer. Upon excitation of the blends at 570 nm, a slightly increased photoluminescent peak is observed, probably due to the interaction of quinoline-pyridine block with the PC_71_BM, reducing the quenching phenomenon of the initial donor:acceptor system.

We also chose representative films of blends bearing 10% of the P3,6C(EH)DTBT-PQPy and the P2,7C(HD)DTBT-PQPy copolymer (blend 3 and 6), which were annealed at 80 °C in order to investigate potential differences in their optical properties. In any case, no significant effect was observed.

**Figure 7:**
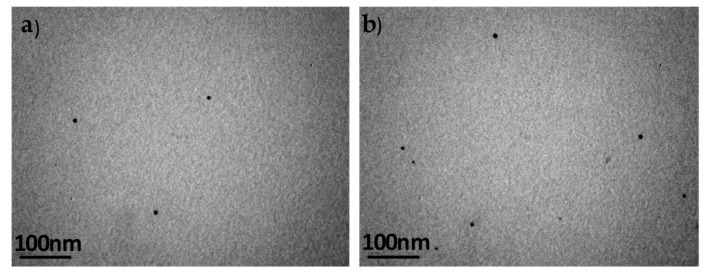
TEM images of a PCDTBT:PC_71_BM 1:1 w/w (**a**) before and (**b**) after annealing at 80 °C. The scale bars correspond to 100 nm.

Thin film morphology of the prepared blends was investigated using transmission electron spectroscopy (TEM), and the images for the blends of PCDTBT:PC_71_BM with the PCDTBT-PQPy copolymers are presented in this section. PC_71_BM has higher electron density compared to PCDTBT, so electrons are scattered more efficiently from the TEM beam. Thus, the darker regions in the TEM images are regions of phase-separated PC_71_BM. In Figure 7, TEM images of blend 1 (PCDTBT:PC_71_BM blend) before (Figure 7a) and after (Figure 7b) annealing at 80 °C, which is a typical temperature used across the literature for such systems, are presented. A uniform, phase-separated at the nanometer scale morphology is observed, without aggregates, in agreement with literature results for the same system (PCDTBT:PC_71_BM 1:1 from *o*-DCB solvent) [49].

**Figure 8:**
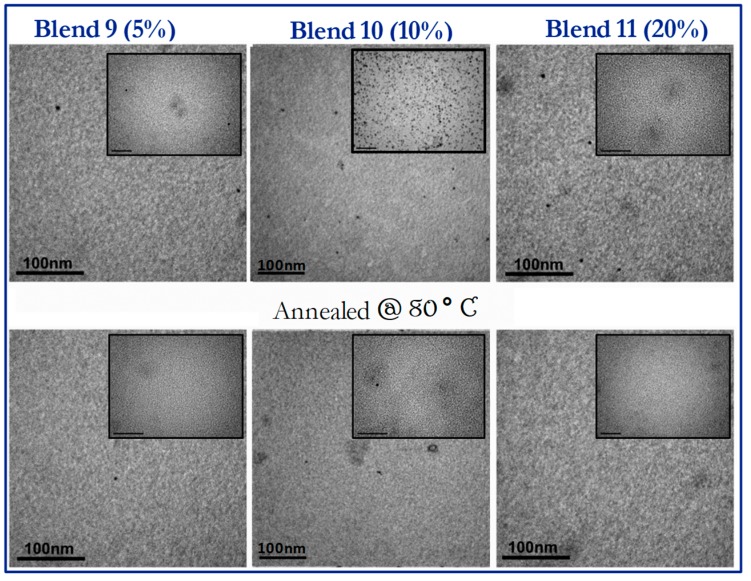
TEM images of a blends 2–4 (PCDTBT:PC_71_BM 1:1 w/w containing 5%, 10% and 20% wt of P3,6C(EH)DTBT-PQPy without annealing (top images) and after annealing at 80 °C (bottom images). For the bigger images, the scale bars correspond to 100 nm, while for the inset images, they correspond to 50 nm.

In Figure 8, TEM images for blends 2–4, bearing 5%, 10% and 20% of the P3,6C(EH)DTBT-PQPy copolymer, respectively, before and after annealing at 80 °C are presented. For blends 2 and 4, bearing 5% and 20% of the P3.6C(EH)DTBT-PQPy copolymer, respectively, no alternations are observed before and after annealing. In the case of blend 3, the formation of a homogeneous thin film is observed for the as-drop-casted film. In some areas of the film, and at higher magnifications, some spherical domains are apparent, as depicted in the inset TEM image. After thermal annealing of blend 3, those spherical domains are no longer detectable. In blend 4, which contains 20% copolymer, uniform nanophase-separated films are formed, whereas after thermal annealing the morphology of the film is preserved.

**Figure 9:**
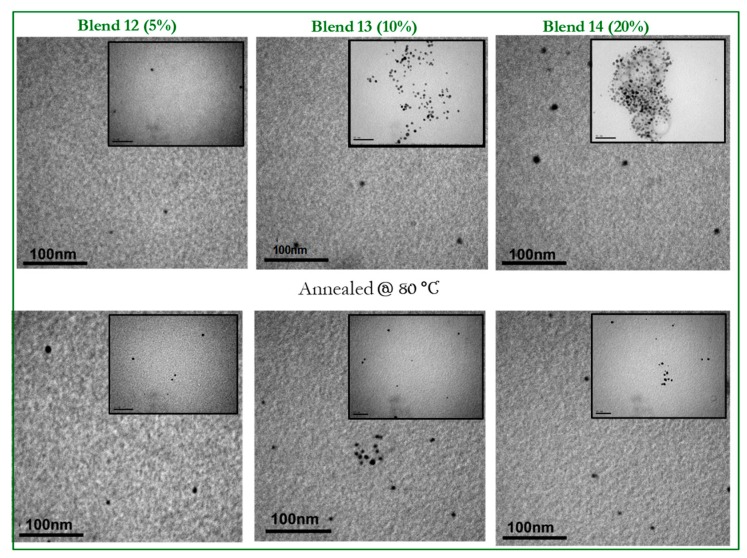
TEM images of blends 5–7 (PCDTBT:PC_71_BM 1:1 w/w containing 5%, 10% and 20% wt of P2,7C(HD)DTBT-PQPy without annealing (top images) and after annealing at 80 °C (bottom images). For the bigger images, the scale bars correspond to 100 nm, while for the inset images, they correspond to 50 nm.

In Figure 9, the TEM images for blends 5–7, bearing 5%, 10% and 20% of the P2,7C (HD)DTBT-PQPy copolymer, respectively, are depicted, before and after annealing at 80 °C. Homogeneous thin films with a more intense nanophase-separated morphology compared to the blends bearing the P3,6C(EH)DTBT-PQPy copolymer are observed for the as-drop-casted films. Blend 5, bearing 5% of the P2,7C(HD)DTBT-PQPy copolymer, produced uniform thin films with extended phase separation that becomes more pronounced after annealing at 80 °C, where some spherical domains are formed. When the copolymer ratio is increased to 10% and 20% (blends 6 and 7), the films remain uniform and the “spheres” are more detectable. After annealing of the blends, the uniformity of the films is preserved, while the number and size of the “spherical” domains decreases. 

### 3.5. Hybrid Materials from the PCDTBT-P5FQ Copolymer

**Scheme 3:**
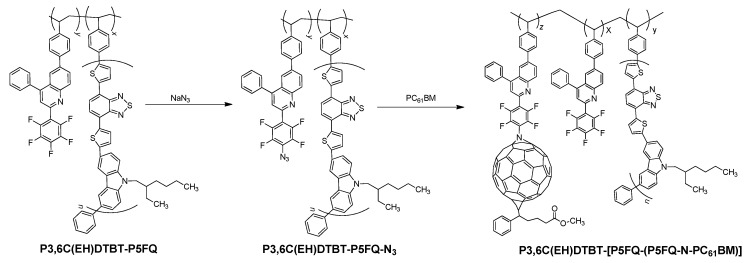
Synthesis of hybrid material P3,6C(EH)DTBT-(P5FQ-(P5FQ-N-PC_61_BM)).

The successful synthesis of copolymers consisting of semiconducting PCDTBT and perfluorophenyl-quinoline blocks opens the route for creating hybrid materials to be used as additives in the active layer of OPVs comprising PCDTBT:fullerene active layers, possibly improving the thin layer’s nanostructure and stability. Thus, the perfluorophenyl-quinoline units of the P3,6C(EH)DTBT-P5FQ copolymers were transformed to azides using sodium azide in THF (Scheme 3) [26,27,28,35]. Successful azidation was confirmed using ATR spectroscopy (Figure 11a), where an intense peak around 2100 cm^−1^ attributed to the azide formation was observed. Thereafter, the azides were employed in a (3+2) cycloaddition reaction with PC_61_BM to provide hybrid semiconducting copolymers. When the perfluorophenyl-quinolines were azidated to a 100% degree, the final hybrid copolymer was insoluble in common organic solvents. To avoid such insolubility problems, we performed the azidation to a 30% degree of the perfluorophenyl groups. At this percentage, the obtained hybrid semiconducting copolymers (Scheme 3) were processable in organic solvents, thus enabling their property characterization.

**Figure 10:**
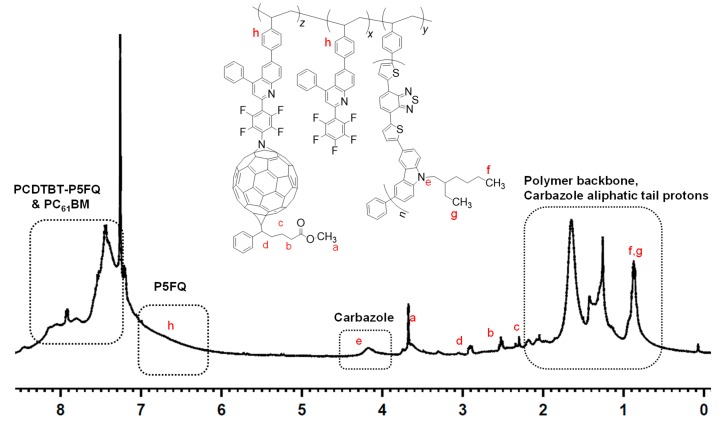
^1^H NMR spectrum of P3,6C(EH)DTBT-(P5FQ-(P5FQ-N-PC_61_BM)) in CDCl_3_.

After the cycloaddition reaction, the obtained hybrid materials were precipitated, redissolved and precipitated again several times in hexane to remove any unreacted PC_61_BM, which was verified each time by TLC using toluene as the mobile face. The ^1^H NMR spectrum of the hybrid copolymer shown in Figure 10 contains the characteristic proton peaks of PC_61_BM (a–d), together with those of the quinoline units at 6.5–7.25 ppm and of the carbazole moieties at 4.2 ppm, confirming the coexistence of all desired semiconducting and fullerene blocks in the final P3,6C(EH)DTBT-(P5FQ-(P5FQ-N-PC_61_BM)) hybrid.

**Figure 11:**
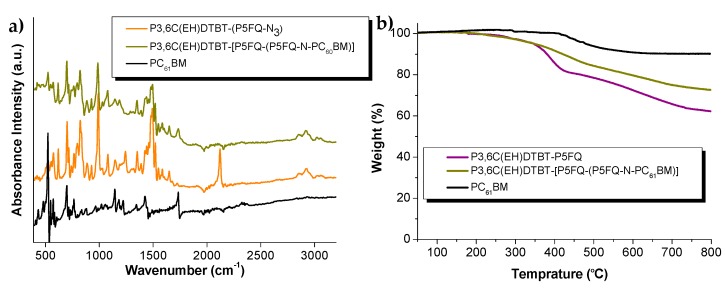
(**a**) ATR spectra of P3,6C(EH)DTBT-P5FQ, P3,6C(EH)DTBT-(P5FQ-N_3_) and P3,6C(EH)DTBT-(P5FQ-(P5FQ-N-PC_61_BM)); (**b**) TGA thermograms of P3,6C(EH)DTBT-P5FQ, P3,6C(EH)DTBT-(P5FQ-(P5FQ-N-PC_61_BM) and PC_61_BM.

Additionally, a thorough ATR investigation (Figure 11a) of the purified hybrid material versus the initially obtained crude product of the cycloaddition reaction and the starting copolymer and fullerene precursors, was carried out in order to assess the efficiency of the purification procedure. The hybrid material presented characteristic peaks at 1735, 1428, 1180, 575 and 526 cm^-1^, which were attributed to the PC_61_BM moieties. During the sequential purification steps of the hybrid copolymer, the ATR spectrum of the intermediates was recorded, and when no further reduction of the fullerene peaks could be observed, we concluded that all unreacted fullerene traces had been successfully removed. The TLC and ATR examination of the intermediates during their purification and of the final hybrid complementarily provided evidence and proof of the hybrid copolymer purity.

Thermogravimetric analysis TGA (Figure 11b) of the initial copolymer and its hybrid derivative with PC_61_BM, P3,6C(EH)DTBT-(P5FQ-(P5FQ-N-PC_61_BM) revealed the anticipated higher carbon residue of the hybrid material compared to the initial copolymer. At 800 °C, a 72.35% residue for the P3,6C(EH)DTBT-(P5FQ-(P5FQ-N-PC_61_BM)) was obtained, whereas a 62.3% residue was obtained for the initial P3,6C(EH)DTBT-P5FQ. From the TGA, an estimate of 36 % wt of fullerene is incorporated in the hybrid derivative which is in a good agreement with the percentage calculated from the NMR (34,7%).

### 3.6. Optical Properties and Morphology Characteriaztion of Hybrid-Material 

**Figure 12:**
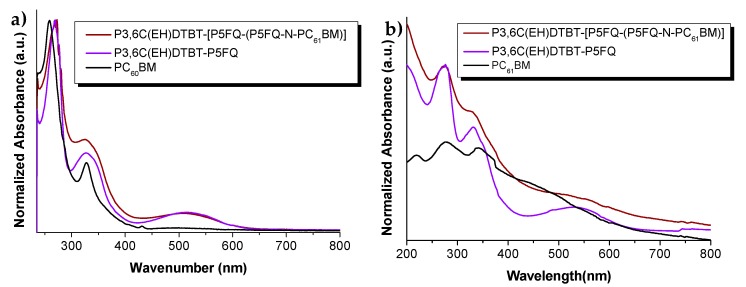
Normalized absorption spectra of P3,6C(EH)DTBT-P5FQ, PC_61_BM and of their hybrid P3,6C(EH)DTBT-(P5FQ-(P5FQ-N-PC_61_BM)) (**a**) in *o*-DCB solutions; and (**b**) in thin film form.

The optical properties of the PCDTBT-P5FQ hybrid with PC_61_BM were examined in comparison to the starting copolymer and fullerene materials. In Figure 12, the normalized UV-Vis absorbance spectra of the P3,6C(EH)DTBT-P5FQ and its hybrid derivative, P3,6C(EH)DTBT-(P5FQ-(P5FQ-N-PC_61_BM), are presented. The UV-Vis spectra of the hybrid are a sum of the net counterparts’ individual spectra, both in solution and in film form. 

**Figure 13:**
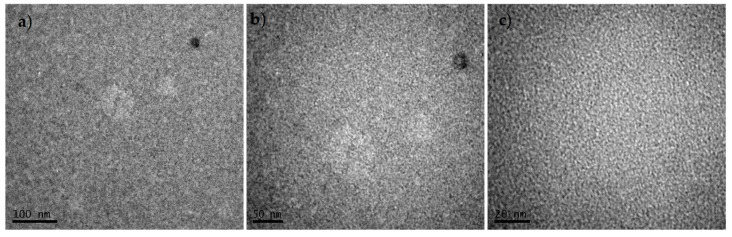
TEM images of P3,6C(EH)DTBT-(P5FQ-(P5FQ-N-PC_61_BM)). The scale bars correspond to 100, 50 and 20 nm for figures (**a**), (**b**) and (**c**), respectively.

Thin film morphology of the P3,6C(EH)DTBT-(P5FQ-(P5FQ-N-PC_61_BM) was investigated using transmission electron spectroscopy (TEM), and the images are presented in Figure 13a–c, where scale bars correspond to 100, 50 and 20 nm, respectively. The TEM images of the hybrid copolymer using *o*-DCB, without any thermal treatment of the sample, present smooth, uniform films of favorable nano-scaled morphology without any fullerene aggregates.

## 4. Conclusions

In summary, a typical electron donor PCDTBT, using either the more soluble 3,6-ethylhexyl carbazole or the 2,7-heptadecanyl carbazole group, has been efficiently modified at the ω-end position with double bonds. A modified procedure was employed in order to control the molecular weight, leading to vinyl-functionalized oligomers of PCDTBT that are soluble enough to be used as macromonomers in free radical polymerizations. The vinyl-functionalized macromonomers were copolymerized either with the vinyl quinoline-pyridine monomer, which can interact with fullerene units via non covalent interactions, or with the vinyl perfluorophenyl-quinoline monomer, which can covalently react via (3+2) cycloaddition with fullerene species. The copolymers presented combined optical properties of the two blocks (PCDTBT and quinoline derivative). The influence of the copolymers on the optical properties of electron-donor:fullerene-acceptor mixtures (PCDTBT:PC_71_BM) was also investigated. The morphology of copolymers and hybrids was investigated using transmission electron microscopy (TEM). The copolymers M1 and M2 were added in a typical active layer blend, PCDTBT:PC_71_BM, in various ratios. Homogeneous thin films were formed, although a more nanophased separated film morphology was observed for the blends bearing the P2,7C(HD)DTBT-PQPy (M2) copolymer. The hybrid material P3,6C(EH)DTBT-(P5FQ-(P5FQ-N-PC_61_BM)) also formed uniform, nanophased phase-separated film. Based on these results, it can be concluded that the particular “end functionalization” of a well-studied electron donor, such as PCDTBT, is conveniently applicable for the development of functionalized semiconducting electron donors. Reacting these materials with other monomers allows the synthesis of new copolymers and hybrid materials with combined properties, which could be employed as additives of typical polymer-donor:fullerene-acceptor active layer blends for OPVs.

## Figures and Tables

**Table 1:** Theoretical and Experimental Ratio of PCDTBT:Quinoline for the copolymers **M1**–**M5**.

Copolymer	PCDTBT-Quinoline Theoretical wt% Ratio	Peak Integrals of ^1^H NMR		molar % Ratio Calculated from ^1^H NMR	wt. % Ratio Calculated from ^1^H NMR
		6.95–6.3 ppm 2H-PQuinoline	4.3–4.1 ppm 2H-carbazole		
**M1**	11/89	3.778	0.022	5/95	7/93
**M2**	11/89	0.555	0.026	5/95	9/91
**M3**	11/89	5.690	1.000	15/85	24/76
**M4**	11/89	0.352	0.090	20/80	23/77
**M5**	11/89	0.700	0.040	5/95	6/94

**Table 2:** Feed ratio and GPC results for the copolymers P3,6C(EH)DTBT-PQPy P2,7C(HD)DTBT-PQPy and P3,6C(EH)DTBT-P5FQ at 254 and 540 nm.

Copolymer	Mn PCDTBT	PCDTBT/Quinoline % molar ratio via ^1^H NMR	GPC @ 254 nm			GPC @540 nm		
			*Mn*	*Mw*	*PDI*	*Mn*	*Mw*	*PDI*
	**PQPy-based copolymers**							
P3,6C(EH)DTBT-PQPy (**M1**)	2100	5/95	3500	4100	1.17	3650	4000	1.1
P2,7C(HD)DTBT-PQPy (**M2**)	3300	5/95	3450	4000	1.16	3500	4000	1.14
P2,7C(HD)DTBT-PQPy (**M3**)	5700	15/85	6000	7000	1.17	5700	6600	1.16
	**P5FQ-based copolymers**							
P3,6C(EH)DTBT-P5FQ (**M4**)	2100	20/80	4900	6100	1.2	5600	6700	1.2
P3,6C(EH)DTBT-P5FQ (**M5**)	2800	5/95	8200	12700	1.55	7700	12000	1.56

**Table 3:** Composition of the different blends studied.

Blend	PCDTBT:PC_71_BM wt. %	Copolymer wt. %
*P3,6C(EH)DTBT-PQPy (M1)*		
**8**	100	0
**9**	47,5:47,5	5
**10**	45:45	10
**11**	40:40	20
*P2,7C(HD)DTBT-PQPy (M2)*		
**12**	47,5:47,5	5
**13**	45:45	10
**14**	40:40	20

## Supplementary Materials

The following are available online at http://www.mdpi.com/2079-4991/9/2/133/s1, Table S1: PCDTBT copolymers nomenclature. Table S2: Preparation of functional PCDTBT oligomers, monomers and conditions employed. Table S3: Abbreviations. Figure S1: ATR spectra of commercial PCDTBT, P3,6C(EH)DTBT-vinyl and P2,7C(HD)DTBT-vinyl CB fractions of the functionalized polymers. The asterisks indicate the ω-end double bonds. Figure S2. ATR spectra of PQPy, P3,6C(EH)DTBT- vinyl and P3,6C(EH)DTBT-PQPy copolymer. Figure S3: GPC Characterization of PCDTBT-P5FQ copolymer and vinyl PCDTBT using the two detectors at 254 and 540 nm. Figure S4: Normalized Absorbance spectra of vinyl PCDTBT macromonomers in o-DCB solutions.
