# Supplementary material for: Copolymers and Hybrids Based on Carbazole Derivatives and Their Nanomorphology Investigation
Source: Nanomaterials (Basel). 2019 Jan 22;9(2):133. doi: 10.3390/nano9020133 (PMC6409860; doi:10.3390/nano9020133)
Supplement: Supplementary file 1 [file nanomaterials-09-00133-s001.pdf]

nanomaterials

Electronic Supplementary Information Copolymers and hybrids based on carbazole derivatives and their nanomorphology investigation

Stefania Aivali[1], Sofia Kakogianni[1], Charalampos Anastasopoulos[1], Aikaterini K. Andreopoulou[1,2] and Joannis K. Kallitsis [1,2,*]

1 Department of Chemistry, University of Patras, University Campus, Rio-Patras, GR26504, Greece; aivali.s@upnet.gr (S.A.); skakogianni@upatras.gr (S.K.); xanastasop@upatras.gr (C.A.); andreopo@upatras.gr (A.K.A.)
2 Foundation for Research and Technology Hellas/Institute of Chemical Engineering Sciences (FORTH/ICE-HT), Platani Str., Patras, GR26504, Greece
* Correspondence: j.kallitsis@upatras.gr; Tel.: +302610-962957

Experimental procedures

Synthesis of end vinyl functionalized P3,6C(EH)DTBT

In a 50mL flame dried and filled with argon round bottom flask, equipped with a reflux condenser and a magnetic stirrer, 4,7-di-(5-bromothiophene-2-yl-2,1,3-benzothiadiazole (1.96 g, 4.28 mmol), 3,6-bis(4′,4′,5′,5′-tetramethyl-1′,3′,2′-dioxaborolan-2′-yl)-N-9″-(ethylhexyl)carbazole (2.50 g, 4.71 mmol) and $Pd(PPh_3)_4$ (0.039 g, 0.034 mmol) were dissolved in 12.5 mL of toluene and refluxed for 20 min under Ar. Then 20% aqueous tetraethylammonium hydroxide solution (2.67 g, 18.15 mmol) imported with a syringe and the mixture refluxed for another 24 h. To introduce the functional groups in the end position of the polymer's backbone 4-vinylphenylboronic acid (0.26 g, 2.14 mmol) was added to the reaction. After 5 h, bromobenzene (0.47 g, 3.00 mmol) was added and the reaction refluxed for 4 h. The mixture was precipitated in cold methanol, filtrated and the received solid was further purified by Soxhlet extraction using sequentially acetone, cyclohexane, tetrahydrofuran. In order to have fraction with higher Mw and in the same time to keep the vinyl end, the polymer was finally washed with chlorobezene at 60°C under Ar for 6 h and filtrated at 60°C. The obtained solid was dried under vacuum at 40°C overnight. Yield: 50%

Synthesis of end vinyl functionalized P2,7C(HD)DTBT

In a 50mL flame dried and filled with argon round bottom flask, equipped with a reflux condenser and a magnetic stirrer, 4,7-di-(5-bromothiophene-2-yl-2,1,3-benzothiadiazole (0.99 g, 2.17 mmol), 2,7-di(4,4,5,5-tetramethyl-1,3,2-dioxaborolan)-N-heptadecan-9-yl-carbazole (di-boronic ester-HD-carbazole (1.50 g, 2.28 mmol) and $Pd(PPh_3)_4$ (0.020 mg, 0.017 mmol) were dissolved in 37.9 mL of toluene and refluxed for 20 min under Ar. Then 20% aqueous tetraethylammonium hydroxide solution (1.36 g, 9.20 mmol) imported with a syringe and the mixture refluxed for another and 16 h. Furthermore, 4-vinylphenylboronic acid (0.13 g, 1.086 mmol) was added to the reaction and after 5 h, bromobenzene (0.24 mg, 1.52 mmol) was added. The reaction refluxed for other 4 h. The mixture was precipitated in cold methanol, filtrated and the received solid was further purified by Soxhlet extraction using sequentially acetone, cyclohexane, tetrahydrofuran. In order to have fraction with higher Mw and in the same time not to damage the the vinyl end, the polymer was washed with chlorobezene at 60 °C under Ar for 6 h and filtrated at 60°C. The obtained solid was dried under vacuum at 40°C overnight. Yield: 50%

Synthesis of PCDTBT-Quinoline copolymers

Table S1. PCDTBT copolymers nomenclature.

PCDTBT oligomers	Quinoline derivative	Solvent	Copolymer
P3,6CDTBT vinyl	vinyl- 5FQ	THF/DMF	P3,6CDTBT-P5FQ
P3,6CDTBT vinyl	vinyl-QPy	THF/DMF	P3,6CDTBT-PQPy
P2,7CDTBT vinyl	vinyl-QPy	THF/DMF	P2,7CDTBT-PQPy

Synthesis of the P3,6C(EH)DTBT-PQPy copolymer

In a 50 mL flame dried round bottom flask, equipped with a reflux condenser and a magnetic stirrer, ω-vinyl end functionalized P3,6C(EH)DTBT-vinyl (0.050 g, 0.066 mmol), 6-vinylphenyl -(2-pyridinyl)-4-phenyl-quionoline (vinyl-QPy) (0.4 g, 1.04 mmol) and azobisisobutyronitrile (AIBN) (0.017 g, 0.104 mmol) were dissolved in 10 mL THF(dry) and 8 mL dimethylformamide. The flask was degassed and flushed with argon again. The reaction mixture stirred at 110°C for 72 h. After evaporation of the solvent, the mixture was precipitated into methanol, filtrated and the obtained solid was washed with diethyl ether and ethylacetate to remove any unreacted monomers. The solid was dried under vacuum at 40°C overnight. Yield 45%

Synthesis of the P2,7C(HD)DTBT-PQPy copolymer

In a 50 mL flame dried round bottom flask, equipped with a reflux condenser and a magnetic stirrer, ω-vinyl end functionalized P2,7C(HD)DTBT-vinyl (0.050 g, 0.0567 mmol), 6-vinylphenyl-(2-pyridinyl)-4-phenyl-quionoline (vinyl-QPy) (0.40 g, 1.04 mmol) and azobisisobutyronitrile (AIBN) (0.017 g, 0.104 mmol) were dissolved in 10 mL THF(dry) and 8 mL dimethylformamide . The flask was degassed and flushed with argon again. The reaction mixture stirred at 110°C for 72 h. After evaporation of the solvent, the mixture was precipitated into methanol, filtrated and the obtained solid was washed with diethyl ether and ethylacetate to remove any unreacted monomers. The solid was dried under vacuum at 40°C overnight. Yield 36%

Synthesis of the P3,6C(EH)DTBT-P5FQ copolymer

In a 50 mL flame dried round bottom flask, equipped with a reflux condenser and a magnetic stirrer, ω-vinyl end functionalized P3,6C(EH)DTBT-vinyl (0.050g, 0.066 mmol), 6-vinylphenyl-(2-perfluorophenyl)-4-phenyl-quinoline (vinyl-5FQ) (0.40 g, 0.845 mmol) and azobisisobutyronitrile (AIBN) (0.014 g, 0.0845 mmol) were dissolved in 10 mL THF(dry) and 8 mL dimethylformamide. The flask was degassed and flushed with argon again. The reaction mixture was stirred at 110°C for 72 h. After evaporation of the solvent, the mixture was precipitated into methanol, filtrated and the obtained solid was washed with diethyl ether and ethylacetate to remove any unreacted monomers. The solid was dried under vacuum at 40°C overnight. Yield: 25%.

Synthesis of [P3,6CDTBT-[P5FQ-P5FQ-N_3]

In a 50mL dried and filled with argon round bottom flack, equipped with a reflux condenser and a magnetic stirrer, the copolymer P3,6CDTBT-P5FQ (0,24 mg, 0.509 mmol) was dissolved in 15 mL THF and 1 mL dimethylformamide (dry) and then NaN_3 (0,008 mg, 0.122mmol) were added. The mixture was degassed, flushed with argon and allowed to heat at 60 °C for 48 h. After the reaction was cooled at room temperature, the mixture was evaporated at reduced pressure in rotary evaporator. The obtained solid was washed several times with deionized water. The final solid was dried under vacuum at 40 °C for 24 h. Yield: 90%

Synthesis of the hybrid analogue of PCDTBT-P5FQ copolymer: [P3,6CDTBT-[P5FQ-(P5FQ-N-$PC_{61}BM$)]

In a 50mL dried and filled with argon round bottom flack, equipped with a reflux condenser and a magnetic stirrer, $PC_{61}BM$ (0.029 g, 0.0314 mmol), the copolymer P3,6C(EH)DTBT-(P5FQ-N_3) (0.05 g, 0.0314 mmol), and 20 mL 1,2-ortho-dichlorobenzene (*o*-DCB) were added and the mixture was degassed and allowed to heat at 160°C for 72 hours. After the mixture was cooled at room temperature, was precipitated in cold hexane and filtered. The solid was evaluated via Thin Layer

Chromatography (TLC), using toluene as the mobile phase on silica gel plates, and ATR for traces of unreacted $PC_{61}BM$ and if necessary the solid was again stirred in toluene. The final hybrid material was dried under vacuum at 40°C for 48 h to give 40 mg of P3,6C(EH)DTBT-P5FQ-(P5FQ-N-$PC_{61}BM$).

Blend Preparation

Stock solutions of commercially available PCDTBT and $PC_{71}BM$ and of P3,6C(EH)DTBT-PQPy and P2,7C(HD)DTBT-PQPy synthesized copolymers were prepared in *o*-DCB (10 mg·mL^{-1}). From these solutions, blends of PCDTBT bearing different ratio of P3,6C(EH)DTBT-PQPy and P2,7C(HD)DTBT-PQPy copolymers (5%,10% and 20% wt of each copolymer) were prepared Afterwards, a mixture of PCDTBT:$PC_{71}BM$ (1:1 wt% ratio) was prepared and P3,6C(EH)DTBT-PQPy and P2,7C(HD)DTBT-PQPy copolymers were added in 5%, 10% and 20% wt ratio.

Table S2. Preparation of functional PCDTBT oligomers, monomers and conditions employed.

Benzothiadiazole monomer	Carbazole monomer	Reaction Time	End capping	Final polymer
4,7-di(2′-bromothien-5′-yl)-2,1,3-benzothiadiazole	3,6-bis(4′,4′,5′,5′-tetra methyl-1′,3′,2′-dioxaborolan-2′-yl)-N-9″-(2-ethylhexyl) carbazole	24h	4-bromophenyl / 4-vinylphenyl boronic acid	P3,6(EH)CDTBT-vinyl
	2,7-bis(4′,4′,5′,5′-tetra methyl-1′,3′,2′-dioxaborolan-2′-yl)-N-9″-(2-heptadacanyl) carbazole	16h	4-bromophenyl/ 4-vinylphenyl boronic acid	P2,7(HD)CDTBT-vinyl

Table S3. Abbreviations.

Abbreviation	Full Name
PCDTBT	poly [N-9'-heptadecanyl-2,7-carbazole-alt-5, 5-(4′,7′-di-2-thienyl-2′, 1′, 3′-benzothiadiazole)]
OPVs	Organic Photovoltaics
P3HT	poly(3-hexylthiophene)
PC61BM	[6,6]-phenyl-C61-butyric acid methyl ester
PC71BM	[6,6]-Phenyl-C71-butyric acid methyl ester
IC60BA	indene-C60 bisadduct
QPy	(2 – pyridinyl)- 4 -phenyl-quinoline
5FQ	(2 - perfluorophenyl) – 4 –phenyl quinoline
PCE	Power Conversion Efficiency

Figures

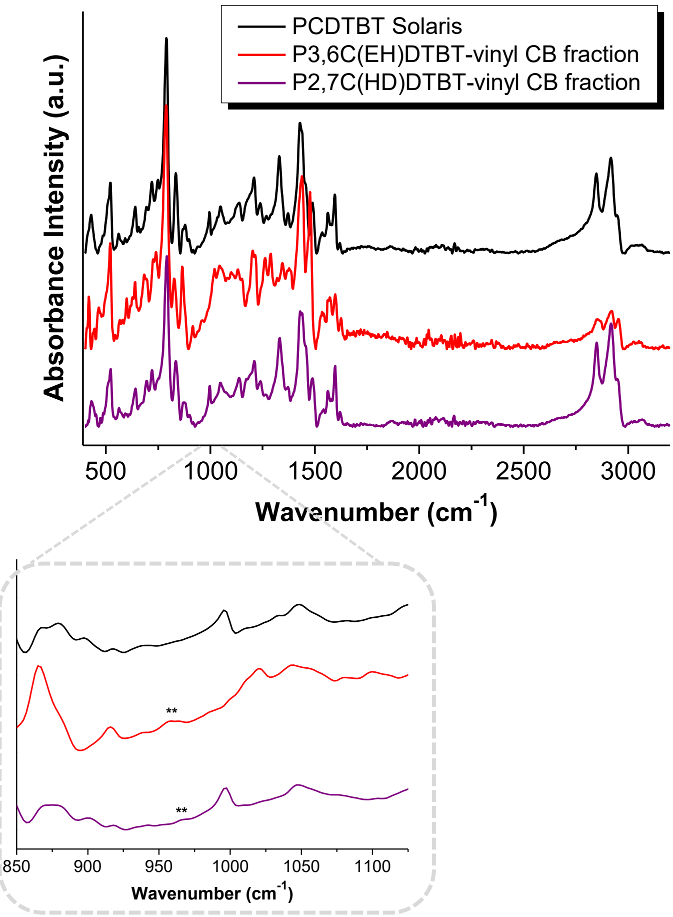

Figure S1. ATR spectra of commercial PCDTBT, P3,6C(EH)DTBT-vinyl and P2,7C(HD)DTBT-vinyl CB fractions of the functionalized polymers. The asterisks indicate the ω-end double bonds.

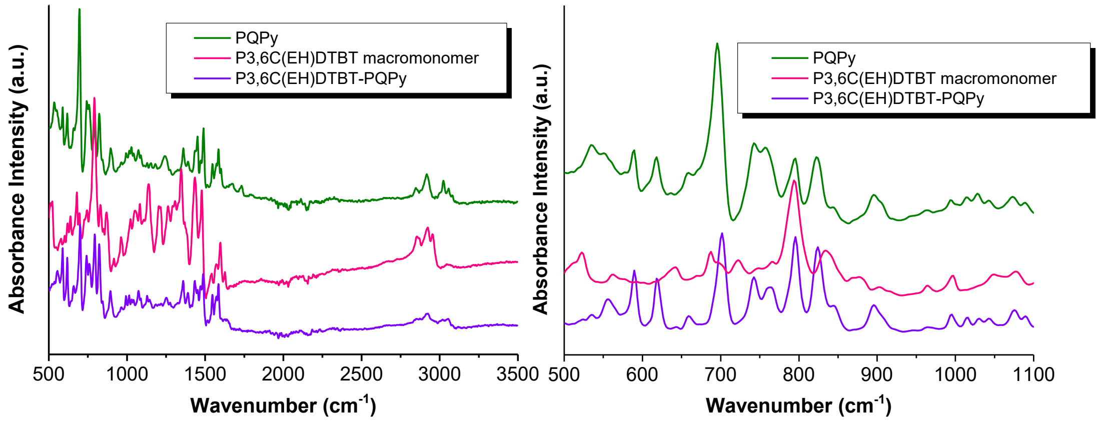

Figure S2. ATR spectra of PQPy, P3,6C(EH)DTBT- vinyl and P3,6C(EH)DTBT-PQPy copolymer.

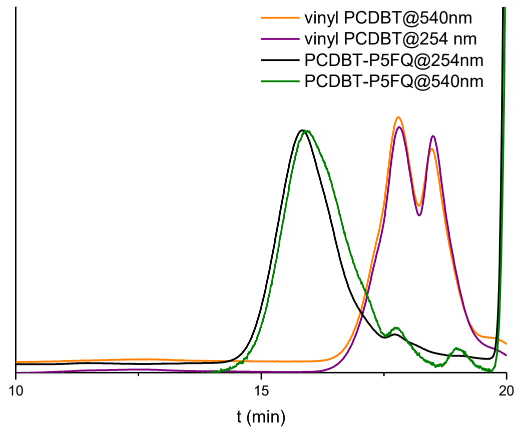

Figure S3. GPC Characterization of PCDTBT-P5FQ copolymer and vinyl PCDTBT using the two detectors at 254 and 540 nm.

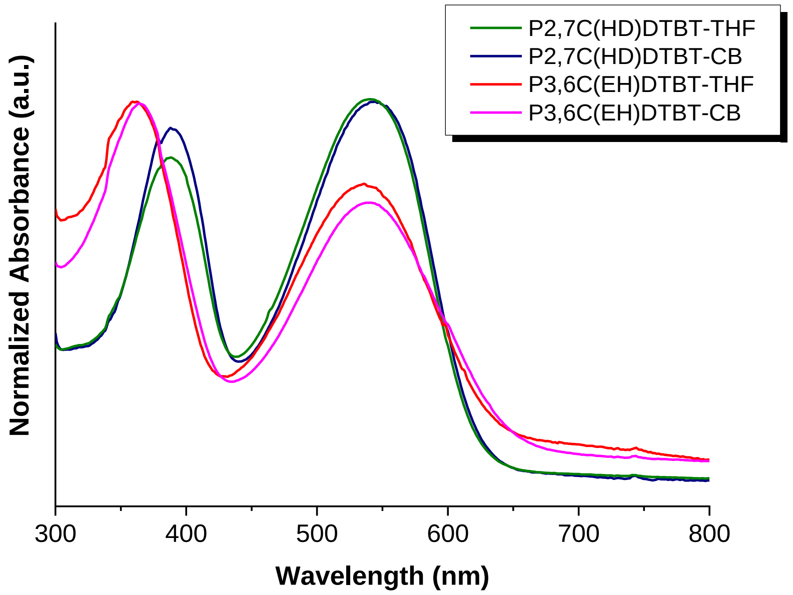

Figure S4. Normalized Absorbance spectra of vinyl PCDTBT macromonomers in *o*-DCB solutions.
